# Editorial: Disclosure Within HIV-Affected Families

**DOI:** 10.3389/fpubh.2018.00140

**Published:** 2018-05-15

**Authors:** Grace Gachanja, Gary J. Burkholder, Aimee Ferraro

**Affiliations:** School of Health Sciences, Walden University, Minneapolis, MN, United States

**Keywords:** child HIV status disclosure, parental HIV status disclosure, HIV disclosure, HIV/AIDS, Sub-Saharan Africa (SSA), resource-poor setting, HIV disclosure process

This special issue, “Disclosure Within HIV-Affected Families,” welcomed manuscripts primarily focused on disclosure of a parent's and/or a child's illness to HIV-positive, negative, and untested children within HIV-affected families. Our goal was to increase the body of knowledge available on how disclosure is performed within these families; however, we did choose to include two manuscripts exploring disclosure among adults.

The majority of the original research was conducted in countries within Sub-Saharan Africa (SSA) where the HIV/AIDS epidemic continues to affect populations disproportionately and where disclosure practices are arguably most critical. The systematic reviews drew from the global published literature on HIV disclosure.

There was a noticeable gap in the literature on disclosure to younger children. In addition, there are few interventions presented that are designed to disclose to younger children. One pervasive assumption is that older adolescents can handle the emotional aspects of disclosure while younger children cannot. Researchers noted in their literature reviews that early disclosure aids in ART adherence and prepares children to protect themselves and their peers as they move into the teenage years when drug and sexual experimentation increases. The research presented suggests that disclosure interventions/practices which train caregivers/parents/partners and healthcare professionals (HCPs) on how to disclose, and then provides post-disclosure support to the persons disclosed to and to the disclosing caregivers/parents/partners, may be important. Summaries of the manuscripts are provided below.

## Original research

Bhatia et al. found that gender inequality in South Africa contributed to fear and mistrust within adult relationships leading to infrequent and complicated partial or implied HIV disclosure. The authors called for integrated interventions aimed at reducing barriers that lead to more trustful and effective communication among HIV-affected men and women.

Chaudhury et al. analyzed the effectiveness of a randomized controlled trial comparing a family-based intervention versus usual social work care to support HIV disclosure among families in Rwanda. Qualitative findings indicated that caregivers and children reported increased stress during the time of disclosure. The authors assert that the family intervention offered structured support for improved parental-child communication which resulted in improved family trust and child mental health.

Cooper et al. documented unexpected shifts in reactions expressed by study participants to their qualitative interviewers over the course of 3 interviews in South Africa. The authors postulated that richer data can be collected with several participant interviews over time as opposed to one-time cross-sectional interviews. During data collection, the possibility of ethical dilemmas arising when participants confide in researchers thereby blurring the researcher's versus a counselor's role during qualitative research was revealed.

Fair et al. found that disclosure is on the mind of HIV-positive parents who were perinatally infected, but they felt their children are still too young to understand the illness. The authors called for additional disclosure training for HCPs who provide adult HIV care because perinatally infected children will eventually transition into their care when they become adults and require future support to disclose to their HIV-positive and negative children.

Namukwaya et al. found that factors motivating disclosure by caregivers in Uganda included curiosity by HIV-positive young people, who reported harboring no resentment despite caregivers' pre-disclosure fears. The authors advocated for disclosure to young people to occur as a planned process with caregivers/parents receiving support from HCPs.

Okawa et al. examined adolescents' perspectives on the best HIV disclosure practices in Zambia, one of the highest HIV-burdened countries in SSA. Adolescents reported being emotionally impacted by disclosure but that it also improved their self-care, adherence to medication, and ability to speak about HIV with their caregivers. The authors called for adolescent post-disclosure support provided by caregivers/parents, HCPs, and peers.

Rochat et al. designed the Amagugu Intervention to enhance the capacity for HIV-infected mothers to disclose their status and educate their young primary school HIV-uninfected children about the disease. A pretest-posttest evaluation of the intervention was found to be acceptable, feasible, and adaptable in other settings. While the authors found that disclosure was not made easier, mothers reported feeling comfortable disclosing to their children and encouraging them to have healthy behaviors.

van Rooyen et al. presented a novel model of successful home-based HIV testing and counseling program with the potential to improve HIV testing, identification of infected family members, disclosure of illness, and linkage to care. The authors addressed implementation challenges that included but were not limited to cultural mores, intergenerational communication among family members, and privacy concerns especially for adolescents.

## Systematic reviews

Aderomilehin et al. conducted a systematic review of SSA literature to determine the perspectives of HCPs and caregivers on disclosure practices to children and adolescents in the region. The authors found that partial disclosure was suitable for children before adolescence and full disclosure was best for adolescents with disclosure being performed by their caregivers with the support of HCPs. The authors recommended family counseling/community education to encourage discussions on sexuality to empower children to make sexual health decisions.

Conserve et al. reviewed the global literature for interventions efficacious in assisting parents living with HIV to disclose their status to their children. Of the five articles from China, South Africa, and the USA, four interventions were found to increase HIV disclosure. Their findings suggested that these effective interventions were adaptable in different cultural contexts/settings.

Krauss et al. reviewed the global literature for reasons used to disclose or not disclose to children. They found that the top three reasons for disclosure were children's curiosity, to improve adherence, and the child's age or maturity level. The top three reasons for not disclosing included fear of the child's negative reaction, young age of the child, and the child's inability to keep the information secret.

Odiachi conducted a literature review on the association between pediatric HIV disclosure and health outcomes in SSA. Five major health outcomes were found including physical/physiological outcomes, psychosocial outcomes, adherence to HIV treatment, sexual and reproductive health, and disclosure of their status to others. The author recommended larger longitudinal studies focused on health outcomes of pediatric disclosure and the creation of policies/guidelines aimed at promoting and improving the low rates of disclosure among this population, especially in SSA.

The HIV disclosure process is known to be difficult and complex. This special topic sheds light on some of the issues that caregivers/parents/partners, children, and HCPs experience before, during, and after HIV disclosure. The manuscript authors suggest feasible interventions and recommendations for future research, in an effort to reduce the HIV disclosure burden within HIV-affected families.

## Author contributions

GG, GB, and AF substantially contributed to the drafting, revising, and final approval of this editorial.

### Conflict of interest statement

The authors declare that the research was conducted in the absence of any commercial or financial relationships that could be construed as a potential conflict of interest.

